# Pesticides and human health

**DOI:** 10.1016/j.jped.2024.11.008

**Published:** 2024-12-24

**Authors:** Marina de Barros Rodrigues, Carlos Augusto Mello da Silva, Débora Carla Chong-Silva, Herberto José Chong-Neto

**Affiliations:** a Departament of Pediatrics, Universidade Federal do Paraná (UFPR), Curitiba, Paraná, Brazil; b Board Certification in Medical Toxicology – Departamento Científico de Toxicologia e Saúde Ambiental da Sociedade Brasileira de Pediatria, Brazil

**Keywords:** Pesticides, Environmental health, Child health

## Abstract

**Objectives:**

to review scientific evidence on the impacts of pesticides on child health, addressing prenatal and postnatal exposures, acute and chronic effects.

**Data source:**

narrative literature review, using databases such as PubMed, SciELO and Google Scholar. The inclusion criteria involved studies published between 2000 and 2023 that analyzed the relationship between pesticides and child health, including systematic reviews, cohort studies, case-control studies and clinical trials. The descriptors used were “pesticides,” “child health,” “prenatal exposure,” “environmental health,” and “developmental toxicity.”

**Data synthesis:**

In this review, an association was observed between exposure to pesticides and the development of neurological and endocrinological diseases, childhood cancer and immunological diseases.

**Conclusions:**

Pesticides represent a significant risk to children's health, with impacts ranging from neurological alterations to chronic diseases. It is essential to promote regulatory changes and encourage agricultural practices that are less dependent on chemical substances, in addition to investing in research that explores long-term impacts and mitigation strategies.

## Introduction

Pesticides are compounds used in agriculture to control pests that affect agricultural food products, such as fruits, vegetables, and green vegetables. Their use in world agriculture started in the 1950s with the aim of increasing food productivity in the post-war years. In Brazil, the term ‘agrochemicals’ is used instead of pesticide, a nomenclature adopted by the Federal Government.^1^ They are classified according to their action target (insecticides, fungicides, herbicides, rodenticides and/or rat poison, acaricides, nematicides, fumigants, molluscicides, defoliants) and according to the chemical group (organophosphates, organochlorines, carbamates, and pyrethroids, among others). They are also classified according to acute and chronic toxicity and hazard to the environment by the Globally Harmonized System of Classification and Labelling of Chemicals (GHS) of the United Nations.^2^

Such compounds pollute the air, water, and soil, persisting in the environment for decades, and affect human health, biodiversity, and ecosystems. Since the late 1990s, concern about the harmful effects of pesticides has been growing globally, with actions being taken by the United Nations Environment Programme (UNEP), leading to the Stockholm Convention in 2001 and the Rotterdam Convention in 2004, which aim to promote the protection of human health and the environment by stopping the use of these compounds.^3^

In Brazil, the use of pesticides has been increasing, markedly from 2016 onwards, with an exponential increase until 2022.^4^ Data released by FAO (Food and Agriculture Organization of the United Nations) show that, in 2021, the country led the use of pesticides worldwide in total volume, 720,000 tons, almost 60 % more than the United States, which is the second largest user in the world, with 457,000 tons. The amount was also higher than that used by China and the United States (USA) together, and the population of China is seven times larger than that of Brazil.^5^ Data from 2022, published in 2024 by FAO, show that Brazil remains in the lead, with 801,000 tons, 70 % more than the USA. Regarding the planted area, Brazil used 12.63 kg of pesticides for each hectare in 2022, whereas the USA used 3.02 kg/ha and China 1.83 kg/ha. Many of these pesticides used in Brazil are highly toxic and have been banned in countries of the European Union, which has the strictest legislation worldwide regarding the use and the allowed limit of residue in water and food.^6^ In Brazil, the maximum allowed limit of glyphosate residue in drinking water is 500 ppm, a value 5000-fold higher than the limit allowed in the European Union.^1^^,^^2^ However, controversially, the European Union is the largest pesticide-producing and exporting region worldwide, supplying highly toxic pesticides to several countries, including Brazil.

According to FAO's 1996 World Food Summit*,* food security is defined as when all people, at all times, have physical and economic access to enough safe and nutritious food to meet their dietary needs and dietary preferences for an active and healthy life.^6^ Therefore, chronic exposure to pesticides through the contamination of food, water, and air puts the entire population in a position of food insecurity. The objective of this review was to expand the knowledge about the effects and action mechanisms of these compounds on human health, especially in the pediatric age group.

## Impact on health

Several diseases and health conditions are associated with exposure to pesticides, including cancer, respiratory diseases, endocrine, metabolic, and cardiovascular alterations. Acute poisonings are easier to identify and usually occur with farmers who handle pesticides. Chronic poisoning, as it results from years of exposure, through the process of bioaccumulation and biomagnification by ingestion of contaminated water and food, is more difficult to identify and can affect the entire population, not only agricultural workers.^6^^,^^7^ Children, as they have less body mass, higher metabolic rate, and are in the full development stage, are more susceptible to the short, medium and long-term effects of pesticides.

There are different types and characteristics of pesticides, with varying exposures and health consequences (Table 1, Table 2).

**Table 1:** Characteristics related to pesticides.

Characteristics of pesticides
Main exposure routes Inhalation: Breathing in pesticide aerosols, vapors, or dust during application or from residues in the air. Dermal Contact: Direct skin contact with pesticides, especially for agricultural workers. Ingestion: Consuming contaminated food, water, or accidental ingestion by children due to improper storage.
Health impacts Acute effects: Short-term exposure to high doses may cause: skin and eye irritation, headache, dizziness, and nausea. Respiratory problems. Severe cases can result in seizures, coma, or death. Chronic effects: Prolonged exposure, even at low levels, can lead to: Cancer: Some pesticides, such as organochlorines, are classified as carcinogenic. - Endocrine disruption: Chemicals such as organophosphates can interfere with hormonal systems. - Neurological disorders: associated with developmental delays, cognitive impairments, and diseases such as Parkinson's. - Reproductive and developmental issues: Potential impacts are infertility, miscarriages, and congenital defects.
Populations at risk Agricultural workers: Often exposed to high concentrations. Children: More vulnerable due to the development of organ systems and greater exposure in relation to body weight. Pregnant women: risk of transmission must be taken into account

**Table 2:** Types of pesticides and the health risks described.

Types of pesticides	Health risks
Organophosphates and Carbamates	Affect the central nervous system (CNS), highly toxic.
Organochlorines	Related to the occurrence of cancer, they persist in the environment.
Herbicides	Irritants to the skin and respiratory system, some of them associated with endocrine disruption.
Fungicides	Potential irritants to the skin and respiratory system.

## Allergic diseases

Asthma is a chronic respiratory disease that affects about 300 million people worldwide, responsible for a thousand deaths every day. Most of these deaths are preventable and occur in low- and middle-income countries. It has a great impact on quality of life and generates expenses for health systems.^8^ There are few studies in the literature on how pesticides can impact the health of the general population, exposed in a non-occupational way, especially studies with children and adolescents. A systematic review with meta-analysis was published in 2022. The analyzed studies involved both postnatal and prenatal exposure and showed that children and adolescents exposed to pesticides have a two-fold higher risk of having asthma symptoms (OR = 2.14; 95 % CI: 1.26–3.64). There was no association between pesticide exposure and allergic rhinitis or atopic dermatitis.^9^

A study carried out in Baltimore, USA, followed 148 children (91 % black and 57 % male) between 5 and 17 years of age diagnosed with asthma for one year. Urine samples were collected every three months and an investigation of asthma symptoms and exacerbations was conducted. They were also submitted to pulmonary function tests and allergic skin tests*.* A positive correlation was shown between higher concentrations of glyphosate and chlorpyrifos biomarkers and cough, wheezing, chest tightness, and respiratory symptoms related to physical exercise. Moreover, increased concentrations of the chlorpyrifos biomarker were associated with a higher risk of hospitalizations.^10^

Another recent study conducted in the USA with almost 2500 children and adolescents aged 6–19 years showed an association between pyrethroid exposure and asthma symptoms in adolescent girls, but not in boys, suggesting a greater susceptibility in the female sex.^11^

A study conducted in Thailand with >300 children showed a positive correlation between exposure to organophosphate pesticides and increased risk of asthma symptoms.^12^ In Italy, >2000 children and adolescents aged between 6 and 14 years from eight cities were evaluated and an increase in ocular, dermatological, respiratory, and systemic symptoms were observed in children exposed to pesticides, according to the residence proximity to the agricultural spraying sites.^13^ Another study conducted in the USA with >700 children between 5 and 12 years old also evaluated the presence of symptoms related to the residence's proximity to agricultural spraying areas. The results were similar to those of the Italian study, with children who lived closer to the cultivation areas with pesticide spraying showing more wheezing in the previous 12 months, as assessed by the ISAAC study.^14^

## Neoplasms

Another important health issue that has also been associated with pesticide exposure is cancer, one of the leading causes of death in children. However, the vast majority of studies have adults as participants, with the greatest exposure being the occupational type. A national study analyzed the extent of water contamination by 11 pesticides with carcinogenic potential in 127 municipalities in the state of Paraná, Brazil. Subsequently, the authors used the cancer risk benchmark concept to estimate cancer cases associated with water contamination by pesticides. Thus, they calculated the probability of an individual exposed to a certain pesticide developing cancer throughout their lifetime. Most of the water samples contained pesticide concentrations above the maximum limits set by the European Union. They estimated 542 cancer cases per million people related to the 11 pesticides analyzed.^15^

A systematic review with meta-analysis that included 28 articles, published between 1966 and 2020, analyzed the relationship between pesticide exposure and brain tumors in children. The studies evaluated prenatal, postnatal, parental occupational, and residential exposure (contact in one's own household or because they lived close to the cultivation area). Prenatal exposure was associated with brain tumor development in children (OR = 1.32; 95 % CI: 1.17–1.49; I^2^ = 41.1 %), as well as postnatal (OR = 1.22; 95 % CI: 1.03–1.45, I^2^ = 72.3 %) and residential (OR = 1.31; 95 % CI: 1.11–1.54, I^2^ = 67.2 %) exposure. Parental occupational exposure was marginally associated with the development of these tumors (OR = 1.17, 95 % CI: 0.99–1.38, I^2^ = 67.0 %).^16^

A case-control study based on records between 1960 and 2015 in Sweden evaluated parental occupational exposure to pesticides and their children's risk of cancer. Maternal occupational exposure was associated with lymphoma (OR = 1.42, 95 % CI 0.78, 2.57) and other solid tumors (OR = 1.30, 95 % CI 0.88, 1.93) in the offspring. No associations were observed between maternal exposure and leukemia or central nervous system tumors. Regarding paternal occupational exposure, an increased risk for myeloid leukemia was observed (OR = 1.15; 95 % CI 0.73–1.79).^17^

## Endocrine disruptors

Pesticide exposure is a risk factor for growth disorders in children living in agricultural areas.^18^ In a case-control study conducted in Thailand, in an area of massive pesticide use, it was observed that children with high pesticide exposure had a risk of stunting of more than three times compared to unexposed children (OR 3.90, 95 % CI 1.15–13.26).

Also in relation to the proximity to agricultural areas, a cohort^19^ carried out in the USA found that pregnant women who lived up to 5 km within the area where the fungicide methyl bromide was used had a higher risk of having fetuses with growth restriction. Exposure during the second trimester of pregnancy was negatively associated with birth weight (β = –113.1 g; CI: 218.1, –8.1), length at birth (β = –0.85 cm; CI: 1.44, –0.27) and head circumference at birth (β = –0.33 cm; CI: 0.67, 0.01).

A cohort conducted^20^ in a district of France between 2004 and 2009 analyzed the concentration of pesticides and nitrates in 1180 water samples and collected data from 11,446 births in 263 municipalities. The authors found an increased risk of small-for-gestational-age (SGA) newborns when pregnant women had been exposed to a mixture of atrazine metabolites and nitrates. The possible mechanism involved is the antiandrogenic action of the mix, which carries an increased risk of SGA newborn.

In another French cohort,^21^ they analyzed 11 pesticides and 29 pesticide metabolites in hair samples from 311 mothers shortly after delivery. The analyses indicated a relationship between exposure to certain organochlorine pesticides and anthropometric measurements at birth (weight, length and head circumference). Higher birth weight was found in the group with intermediate exposure to fipronil sulfone when compared to the group with the lowest exposure level (_adjusted_β = +150 g; 44, 255). Regarding diethyl‑phosphate, lower length at birth was found to be associated with an intermediate level of exposure (_adjusted_β = −0.64 cm; −1.15, −0.14). Longer length at birth was observed in neonates of women with higher concentrations of bioethanol (_adjusted_β = +0.60 cm; 0.09, 1.10) and isoproturon (_adjusted_β = +0.55 cm; 0.11, 1.00) in the hair. Finally, higher head circumference was associated with prochloraz (_adjusted_β = +0.57 cm; 0.17, 0.97) and tebuconazole (_adjusted_β = +0.31 cm; 0.01, 0.61).

A recent systematic review found no consistent association between prenatal pesticide exposure and birth weight and height for any pesticide class. Prenatal exposure to organochlorines is thought to be associated with birth weight; however, the direction of this association remains unclear, with studies showing both positive and negative associations. Additionally, there is no consistent evidence of an association between prenatal pesticide exposure, low birth weight, and preterm birth.^22^ Another meta-analysis found that prenatal exposure to organophosphate pesticides was weakly associated with head circumference at birth, but not with birth weight or length.^23^ Exposure to chlordecone, an organochlorine pesticide, was reportedly not associated with changes in birth weight.^24^

*In utero* exposure to organochlorine pesticides (dichlorodiphenyltrichloroethane [DDT], dichlorodiphenyldichloroethylene [DDE], and hexachlorobenzene [HCB]) may be associated with rapid weight gain in childhood^25^^,^^26^ and later with higher body mass index (BMI)^27^^,^^28^ Positive longitudinal associations have been reported between prenatal exposure to DDT and DDE in children and other obesity-related outcomes.^29^

Prenatal levels of DDE and DDT were significantly associated with increased birth weight of the newborn for both sexes. Exposure to DDE is positively associated with overweight or a high BMI at 6, 12, or 14 months of age.^25^^,^^26^^,^^30^ HCB exposure is significantly associated with increased birth weight, especially in the female sex.^31^

## Neurological disorders

A case-control study investigated the association between children's early exposure to mixtures of non-persistent chemicals and symptoms of Attention Deficit Hyperactivity Disorder (ADHD) in the context of the CHARGE study. Urine samples from 549 children (2–5 years old) diagnosed with Autism Spectrum Disorder (ASD), developmental delay (DD), or typical development (TD) were analyzed. The effects of 62 chemical compounds from four classes (phenols/parabens, phthalates, organophosphate pesticides, and trace elements) on ADHD symptoms measured by the Aberrant Behavior Checklist (ABC) scale were evaluated. Phthalate mixtures, primarily Di(2-ethylhexyl)phthalate (DEHP) and mono-2-heptyl phthalate (MHPP) metabolites were associated with greater symptoms of ADHD (OR = 1.09, 95 % CI: 1.00,1.20), hyperactivity/impulsivity (OR = 1.11, 95 % CI: 1.01, 1.22), and attention deficit (OR = 1.06, 95 % CI: 0.99, 1.13). The associations were stronger in children with ASD. In these children, the overall mix of chemicals was associated with increased ADHD symptoms, most notably some compounds such as phthalates (DEHP, MHPP), one phenol (DHB34), and cadmium (Cd). The pesticide diethyl phosphate (DEP) showed non-significant associations with ADHD (OR = 1.01, 95 % CI: 0.92, 1.09), hyperactivity/impulsivity (OR = 1.02, 95 % CI: 0.93, 1.12), and attention deficit (OR = 1.01, 95 % CI: 0.94, 1.08).^32^

One study evaluated the association between environmental exposure to pesticides and the prevalence of neurodevelopmental disorders in children. It was carried out in the province of Almería, Spain, known for its high concentration of greenhouses and intensive use of pesticides. A total of 4830 children under five years of age diagnosed with neurodevelopmental disorders were included over an 11-year period (2011–2022). The housing areas were classified as high, medium, and low pesticide exposure based on agronomic use. Chromosomal aberrations were the most prevalent prenatal risk factors (28.6 % of cases). Perinatal factors were gestational age <32 wk (25 %), whereas postnatal factors were brain lesions evidenced by neuroimaging (36.7 %). Children from areas with high pesticide exposure had a higher prevalence of disorders and the district of Almeria Oeste, with the highest pesticide use, had a higher incidence (prenatal diagnosis OR = 1.45, 95 % CI: 1.10–1.91 and perinatal diagnosis OR = 1.29, 95 % CI:1.01–1.96). Boys were more vulnerable to neurodevelopmental disorders. The mean age at referral was 2.05 years; younger age was associated with higher risk. The authors suggest that pesticide exposure may negatively impact childhood neurodevelopment, especially in areas with intensive use. Disorders such as developmental delays, autism, and attention deficit disorder may be associated with early pesticide exposure.^33^

## Metabolic and cardiovascular effects

A study of Japanese children analyzed the relationship between LDL cholesterol levels in the blood of pregnant women in the first trimester and the urinary metabolites of organophosphate pesticides (OPP). The study is part of the Japan Environment and Children's Study (JECS) and included 5169 pregnant women. In overweight women, higher levels of the metabolite diethyl phosphate (DEP), an organophosphate pesticide, were significantly associated with elevated LDL cholesterol levels (odds ratio = 1.32). No significant overall associations were found between LDL-C levels and other organophosphate metabolites (DMP, DMTP). BMI modified the effects of urinary metabolites on LDL-C levels, highlighting that being overweight increases susceptibility. There was no significant association of the metabolites with other lipids, such as total cholesterol and HDL. It was concluded that exposure to pesticide metabolites, especially DEP, can affect LDL cholesterol levels in overweight women during pregnancy. Future studies are needed to clarify the underlying biological mechanisms and to explore the impacts on maternal and newborn health.^34^

In another evaluation to verify the association between exposure to organophosphate pesticides during pregnancy and cardiovascular outcomes in children at 10 years of age, concentrations of urinary metabolites of organophosphate pesticides were evaluated in 643 mother-child binomials. Increases in 4-dimethylaminopyridine (DMAP) metabolites were associated with reduced carotid artery elasticity and higher systolic blood pressure, indicators of poorer vascular health. Conversely, increases in diethyl alkyl phosphate (DEAP) metabolites were associated with lower carotid intima-media thickness and higher HDL cholesterol levels, suggesting a protective cardiovascular profile. Specific genotypes of the PON1 gene (such as PON1–161CC and PON1-L55MTT) modified the effects of exposure, with an increased risk of adverse vascular and glycemic outcomes in children. Despite some positive associations, exposures to organophosphate pesticides during pregnancy suggest potential risks to infant cardiovascular health, especially in genetically vulnerable subgroups.^35^

## Regulation and preventive measures

The registration of new pesticides involves evaluation by three federal government agencies: the Ministry of Agriculture, Livestock and Food Supply (MAPA, *Ministério da Agricultura, Pecuária e Abastecimento*), the Brazilian Institute of the Environment and Renewable Natural Resources (IBAMA, *Instituto Brasileiro do Meio Ambiente e dos Recursos Naturais Renováveis*) and the National Health Surveillance Agency (ANVISA, *Agência Nacional de Vigilância Sanitária*), with MAPA being the agency responsible for providing the registration after evaluation by ANVISA and IBAMA.^36^ Aiming at monitoring pesticide residues in foods of plant origin, the Program for the Analysis of Pesticide Residues in Food (PARA, *Programa de Análise de Resíduos de Agrotóxicos em Alimentos*) was created in 2001, coordinated by ANVISA.^37^ In cases of acute poisoning, the nearest Toxicological Information and Assistance Center (CIATox, *Centro de Informação e Assistência Toxicológica*) should be contacted through a 0800 number, which is part of the National Network of Toxicological Information and Assistance Centers (Renaciat, *Rede Nacional de Centros de Informação e Assistência Toxicológica*), created in 2005 and coordinated by ANVISA.^38^ Aiming to mitigate the impact of pesticides on human and children's health, regulatory and preventive measures must be adopted (Table 3).

**Table 3:** Regulatory advances and preventive measures for pesticide use.

Regulatory advances and prevention
Regulatory advances *Global Policy Updates* - The European Union banned chlorpyrifos in 2020 after evidence of its neurotoxic effects. - Brazilian regulatory agencies imposed stricter residue limits in 2022. *Integrated Pest Management (IPM):* Countries such as India and the Philippines have intensified IPM initiatives, promoting biopesticides and cultural practices to minimize the use of synthetic pesticides.
Preventive measures Consumer Actions: The growing awareness of washing produce and choosing organic options has gained strength, supported by studies confirming the reduction of pesticide residues in organic diets. Education and training: Programs targeting agricultural workers and smallholder farmers have successfully reduced the misuse and overapplication of pesticides in regions such as Africa and Latin America.

## Conclusion

Pesticide exposure is a significant public health problem, with evidence associating it with acute and chronic effects on the health of individuals and children are not exempt from these risks, resulting in a great impact on morbidity and mortality. Vulnerable populations, especially children and agricultural workers, need targeted protection. Effective mitigation strategies include strengthened regulations, public awareness campaigns, and sustainable farming practices. It is essential that the toxicological indices (Maximum Residue Limit (MRL), Acceptable Daily Intake (ADI), and Acute Reference Dose (ARfD))^39^ be reviewed and reduced, as has been done in the European Union, aiming to limit exposure to these compounds. Concomitantly, the agencies responsible for supervising their use (PARA/ANVISA) should be strengthened. In parallel, the legislation that provides for the registration of new pesticides and everything involving these compounds should also be reviewed, and a national coalition is therefore necessary to mitigate the effects of acute and chronic exposure on the entire population. For that purpose, it is essential to have more national studies on the subject, which can support national policies aimed at health prevention and promotion, focused on the pediatric age group.

## Authors' contributions

Marina de Barros Rodrigues, Carlos Augusto Mello da Silva, Débora Carla Chong-Silva, Herberto José Chong-Neto: Intellectual author, literature review, data collection, review, and writing of the manuscript.

## Conflicts of interest

The authors declare no conflicts of interest.
